# Bipolar Thermofusion BiClamp 150 in Thyroidectomy: A Review of 1156 Operations

**DOI:** 10.1155/2014/707265

**Published:** 2014-04-16

**Authors:** Tomáš Pniak, Martin Formánek, Petr Matoušek, Karol Zeleník, Pavel Komínek

**Affiliations:** Department of Otolaryngology, University Hospital Ostrava, 17 Listopadu 1790, 708 52 Ostrava, Czech Republic

## Abstract

*Objectives*. To compare the bipolar thermofusion BiClamp 150 with conventional ligature techniques for thyroid gland surgery, and report the advantages/disadvantages of both techniques. *Methods*. In this retrospective comparative study, all thyroid gland operations performed in the ENT Clinic Faculty Hospital Ostrava from 2006 to 2013 were included (1156 operations, 2122 lobes). Patients were categorized into two groups according to the type of vessel sealing method used, group I (BiClamp, *n* = 819 operations) and group II (conventional ligature, *n* = 337 operations). The number of revision surgeries due to wound hematoma was recorded as a bleeding event. Statistical analysis of the complication rate (bleeding rate, recurrent nerve palsy) and time of duration was performed. *Results*. The rate of revision surgery performed due to postoperative wound hematoma was significantly lower in group I (15/819, 1.83%) compared with group II (14/337, 4.15%) (*P* = 0.022). There was no statistically significant difference in the frequency of recurrent laryngeal nerve palsy between groups I and II (*P* = 0.36). The average surgery time was significantly shorter in group I (*P* < 0.001). *Conclusion*. Bipolar thermofusion BiClamp is an effective vessel sealing method that leads to a significant reduction in postoperative wound bleeding rates and reduces surgical time compared with conventional vessel ligature.

## 1. Introduction

The success of thyroid gland surgery depends on meticulous hemostasis in the operative field; otherwise, there is a possibility for numerous complications to occur, ranging from worsened wound healing due to hematoma to massive life-threatening hemorrhage [1]. Recurrent laryngeal nerve (RLN) damage is another possible complication of this type of surgery, occurring in 0.8–2.5% of all lobes operated on according to the literature [1].

Hemostasis during thyroidectomy can be performed by classic suture ligature (clamp-and-tie technique) or by electrocoagulation. Both methods are frequently used to control bleeding [2]. During the past few years, a variety of energy-based techniques for vessel sealing have been introduced [2–5]. For example, there is a harmonic scalpel that uses vibration energy [2]. Another novel technique for vessel sealing is bipolar thermofusion of tissue, for example, the frequently used marketed product, LigaSure [4, 5]. In our department, we use another marketed product BiClamp 150 (ERBE) that is specially designed for thyroidectomies.

The goal of our current study was to compare the bipolar thermofusion system, BiClamp 150, with the conventional clamp-and-tie technique for use during thyroid gland surgery in the terms of its effectiveness in hemostasis, safety in relationship with possible RLN damage, and possible shortening of the operation time.

## 2. Material and Methods

### 2.1. Study Design

During the period from September 2006 to June 2013, we retrospectively evaluated all 1156 thyroid gland operations performed in the ENT Clinic Faculty Hospital Ostrava. All patients who underwent a thyroid procedure were eligible for inclusion. The aim of this study was to compare the effectiveness of hemostasis and safety of the bipolar thermofusion system, BiClamp 150 in thyroid gland surgery with the conventional clamp-and-tie technique. The indications for surgery included all kinds of thyroid pathology (benign and malignant tumors of the thyroid gland and parathyroid tumors with thyroid extension, multinodular goiter, thyroiditis lymphomatosa, and others), including reoperative thyroid surgery due to residual thyroid tissue. Extent of surgery was considered to be total thyroidectomy and hemithyroidectomy, including extirpation of residual thyroid tissue in reoperative surgery. In the case of malignancy with lymph node involvement, block neck dissection was performed concurrently. We also included operations of the parathyroid gland with thyroid extension and hemithyroidectomy performed in our cohort. All operations were performed under general anesthesia, with the patient lying on their back and the head extended backwards. In all patients, before closure negative suction drain was placed and compression bandage was applied. Drain was removed on second postoperative day.

The study was performed in accordance with the Declaration of Helsinki, Good Clinical Practice, and applicable regulatory requirements. Written consent was obtained from all patients before the initiation of surgery.

### 2.2. Division of Patients and Rating

The patients were divided into two groups according to the type of vessel sealing used during the surgery. In group I, the bipolar thermofusion system, BiClamp 150, was used to seal the superior and inferior thyroid vessels and lateral thyroid veins. Conventional bipolar coagulation was used in smaller vessels. In this group, conventional ligature was not used at all. In group II, a conventional clamp-and-tie technique was used for ligating of the superior and inferior thyroid vessels and lateral thyroid veins. Smaller vessels were coagulated using conventional bipolar coagulation.

The number of revision surgeries due to wound hematoma was a parameter of hemostasis effectiveness for each group in our cohort. In this study, perioperative blood loss was not taken as a parameter due to wide spectrum of diagnosis operated. The number of permanent RLN palsies was a parameter of the safety of surgery. Only recurrent palsy lasting more than one year after the surgery was considered in the ratio of the number of palsies to the number of lobe operations. The duration of surgery was evaluated as a parameter of time saving. We did not evaluate possible hypocalcaemia after thyroid gland surgery in this cohort.

### 2.3. Bipolar Thermofusion with BiClamp 150 (ERBE)

**Figure 1:**
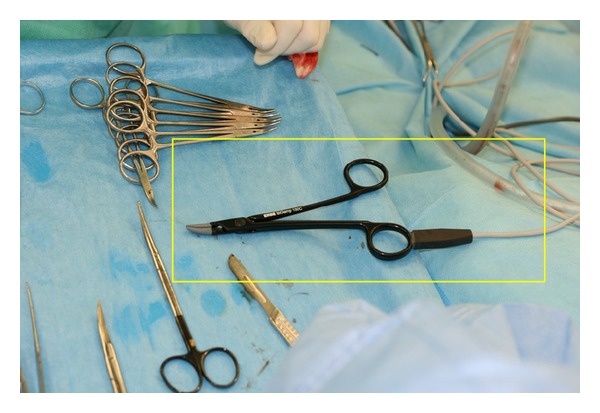
BiClamp manipulator.

Bipolar thermofusion BiClamp 150 (ERBE Elektromedizin GmbH, Teubingen, Germany, www.erbe-med.com) is a tool specifically designed for thyroid surgery. It works on the principle of bipolar thermofusion of tissue. Low-voltage energy is strong enough to seal vessels of up to 7 mm in diameter on the basis of collagen fiber fusion [3]. The handpiece is shaped like forceps and is easily used to grasp and coagulate the vessel. It is useful for tissue preparation as well (Figure 1). After placing the handpiece into the proper position on the vessel and pushing the pedal, electrocoagulation starts. The process ends with acoustic signals from the processing unit, after the vessel is sealed and it is possible to cut it. An automatic stop function prevents heat injury from the surrounding tissues. Another advantage of the instrument is the possibility of resterilization. It should not be used in the area of the RLN due to the potential risk of injuring the nerve from the dispersion of heat. The tool is not suitable for coagulation of very small vessels because forceps tips of BiClamp are thicker than conventional bipolar coagulation tips. Encrusted coagulations could adhere to the forceps tips and there is danger of damaging the vessel when the forceps are withdrawn.

### 2.4. Statistical Analysis

The *χ* ^2^ test for 5% significance was used to analyze differences in revision surgery rates and recurrent nerve palsy rates between groups I and II, with *P* < 0.05 considered statistically significant. For analysis of the duration of the operation, nonparametrical two-sample Wilcoxon rank-sum (Mann-Whitney) test was used for 5% significance. Stata software (version 10) was used for all statistical calculations.

## 3. Results

During the period from September 2006 to June 2013, there were 819 (1522 lobes) thyroid gland surgeries in group I (BiClamp). In group II (conventional ligature), there were 337 thyroid gland surgeries (600 lobes). The male to female ratio was 1/5.4.

In group I, the ratio of revisions to surgery was 15 to 819 (1.83%). In group II, revision due to bleeding was performed 14 times in 337 surgeries (4.15%). The total postoperative bleeding rate was 2.51%, and all revisions were performed within 24 hours after surgery. The ratio of revisions to surgery was significantly lower in group I (BiClamp) compared with surgery with conventional ligature (*P* = 0.022).

RLN palsy was observed in 22/1522 (1.45%) lobes in group I and 12/600 (2.0%) in group II. No significant difference between groups was observed (*P* = 0.36).

**Figure 2:**
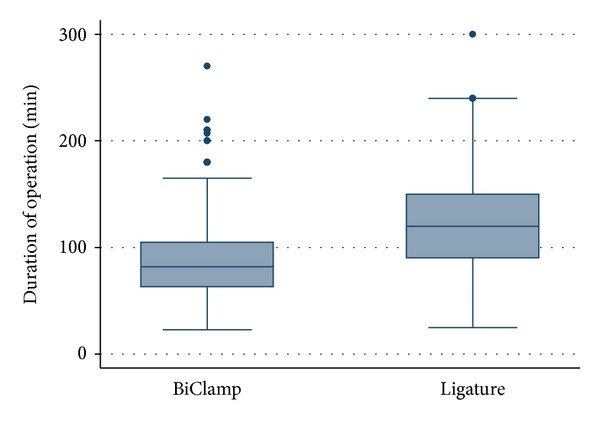
Duration of surgery in minutes for group I (BiClamp) and group II (ligature).

The average ± standard deviation duration of total thyroidectomy was 89.6 ± 27.6 minutes in group I and 122.9 ± 37.7 minutes in group II (Table 1). The average duration of surgery was decreased by 25.99% (Figure 2). The difference in time saving in group I was statistically significant compared with group II (*P* < 0.001).

## 4. Discussion

In the past few years, novel methods of vessel sealing without using conventional ligature have emerged [2–6]. In addition to spray coagulation or harmonic scalpel, there is also the principle of bipolar tissue thermofusion as used by BiClamp 150 [3]. In our department, this system is frequently used in thyroid gland surgery. Traditionally, use of electrosurgical bipolar thermofusion systems spread to head and neck surgery from abdominal surgery and gynecology [4]. A randomized study by Silva-Filho in 45 patients with vaginal hysterectomies showed shorter operative times, faster recovery, lower perioperative blood loss, and less pain with the bipolar vessel sealing system compared with conventional sutures [4]. However, since 2003 several studies have reported successful usage of bipolar vessel sealing systems in thyroidectomy [5–7]. In his comparable study of 155 patients, Franko et al. emphasize that LigaSure bipolar electrosealer, when used as the primary means of hemostasis during thyroidectomy, significantly reduced mean operative times, whereas the rates of perioperative complications were unchanged [5]. Lachanas et al. obtained similar findings during thyroid surgery with LigaSure in 72 consecutive patients. There was a mean reduction in operative time of 23 minutes compared with previous surgical thyroid procedures when a bipolar vessel sealing system was not used [6]. Manouras et al. compared the outcome of thyroidectomy using an electrothermal bipolar vessel sealing system (*n* = 148), the harmonic scalpel (*n* = 144), and classic suture ligation techniques (*n* = 90). Compared with the classic technique, surgical time was reduced by about 20% when a bipolar sealer or harmonic scalpel was used. The 3 groups were similar in terms of perioperative complications, hospital stay, and thyroid gland pathology [7].

Data in the literature show rates of about 1.72–4.2% postoperative bleeding that require revision of thyroidectomy wounds [8–10]. In a study of 30,142 thyroid gland operations, Promberger et al. observed postoperative bleeding in 1.7% [8]. Risk factors identified were older age, male sex, extent of the resection, bilateral procedure, and operations for recurrent disease. The risk of postoperative bleeding doubled during bilateral thyroid surgery compared with unilateral surgery, occurring in 2.0% of bilateral operations compared with 1% of unilateral operations [8]. A high frequency of total thyroidectomy (663/819 in group I, 250/337 in group II) in our cohort is probably one of the factors that influenced the higher frequency of postoperative bleeding (the number of vessels treated is doubled during total thyroidectomy). Morton et al. identified a postoperative systolic blood pressure of greater than 150 mmHg as a major significant factor associated with an increased risk of hemorrhage following thyroid surgery [9]. Finally, the method of vessel sealing influenced the postoperative bleeding rate. Saint Marc et al. in his prospective study of 200 patients found that 1 patient in the LigaSure group (*n* = 100) and 2 patients in the clamp-and-tie group (*n* = 100) required revision surgery due to hematoma. Despite this, the authors considered LigaSure to be as safe as the clamp-and-tie technique [11]. Franko et al. obtained similar results. One patient in each group (LigaSure group, *n* = 85, clamp-and-tie group, *n* = 70) developed neck hematoma requiring surgery [5]. In our cohort, bipolar thermofusion during surgery decreased the frequency of postoperative bleeding during surgery compared with classical vessel ligature (clamp-and-tie method). The frequency of wound revision due to bleeding was significantly lower in the thyroid surgery with use of BiClamp compared with conventional ligature (*P* = 0.022).

We did not observe any statistically significant difference in the frequency of RLN palsies based on the method of vessel sealing (BiClamp, conventional ligature) (*P* = 0.36). BiClamp has not been used in the area of the RLN because it is not recommended by the manufacturer due to possible heat dispersion and injury of the nerve. In this area only conventional bipolar coagulation was used in BiClamp group. The incidence of RLN paresis in the literature is 0.8–2.5% of all lobes operated, with higher rates occurring in older patients [1, 12–14]. Our findings are in agreement with these.

According to the literature, bipolar thermofusion could reduce the duration of surgery [5–7]. Franko et al. observed significantly shorter times when hemostasis was achieved with a bipolar electrosealing device (LigaSure, *n* = 85, 110 ± 33 minutes) compared with conventional ties (*n* = 70, 130 ± 37 minutes, *P* < 0.001) [5]. Manouras et al. found that surgical time was reduced significantly by about 20% when the bipolar vessel sealer or harmonic scalpel was used compared with the classic technique (93 ± 12.5 versus 74.3 ± 14.2 and 73.8 ± 13.8 minutes). Such findings were also shown in our cohort. In group I (BiClamp) we found a 26% time reduction compared with group II (conventional ligature) (*P* < 0.001). However, the BiClamp bipolar vessel sealing system is reusable compared to single-use manipulators such as the harmonic scalpel. This decreases the cost of surgery and may justify use of a bipolar vessel sealing system in ENT departments.

## 5. Conclusion

The bipolar thermofusion BiClamp is an effective and safe method for vessels sealing during thyroidectomy, including sealing of the main thyroid vessels. BiClamp may achieve statistically significant reductions in the frequency of postoperative bleeding compared with conventional vessel ligature. Using bipolar thermofusion also leads to significant reductions in operative time.

## Figures and Tables

**Table 1:** Duration of surgery in minutes according to extent of the surgery.

Group	BiClamp			Ligature			*P* value*
	*N*	Median (min)	Mean (min)	*N*	Median (min)	Mean (min)	
TTE	679	90	89.66	263	120	122.90	<0.001
TTE + BND	26	137.5	145.08	9	165	171.67	0.144
HTE	114	60	63.12	65	75	82.63	<0.001
Total	**819**	**82**	**87.72**	**337**	**120**	**116.44**	**<0.001**

HTE: hemithyroidectomy; TTE: total thyroidectomy; BND: bloc neck dissection.

*Two-sample Wilcoxon rank-sum (Mann-Whitney) test.
